# Supplementary material for: Limited Variation between SARS-CoV-2-Infected Individuals in Domain Specificity and Relative Potency of the Antibody Response against the Spike Glycoprotein
Source: Microbiol Spectr. 2022 Jan 26;10(1):e02676-21. doi: 10.1128/spectrum.02676-21 (PMC8791189; doi:10.1128/spectrum.02676-21)
Supplement: SUPPLEMENTAL FILE 1 — Supplemental material. Download SPECTRUM02676-21_Supp_1_seq4.pdf, PDF file, 0.4 MB [file spectrum02676-21_supp_1_seq4.pdf]

Supplemental Figure S1

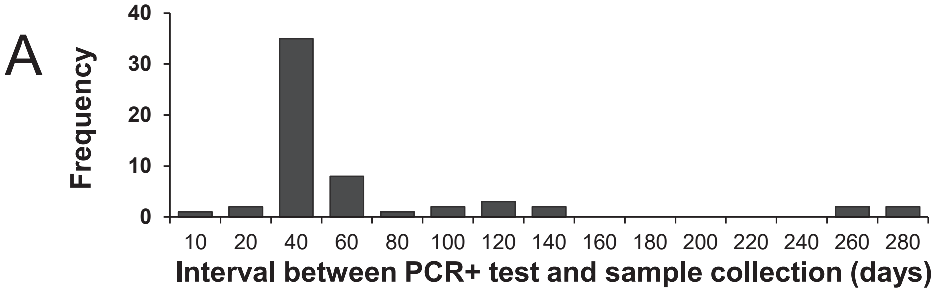

B

	P value	*P* value for log_{10}-transformed data
**NTD**	<0.0001	0.11
**RBD**	<0.0001	0.08
**Ecto**	<0.0001	0.29
**Liaison**	<0.0001	0.56
**Ortho**	0.82	<0.0001
**TrimericS**	<0.0001	0.67
**Roche**	<0.0001	<0.0001
**cbELISA**	<0.0001	0.76

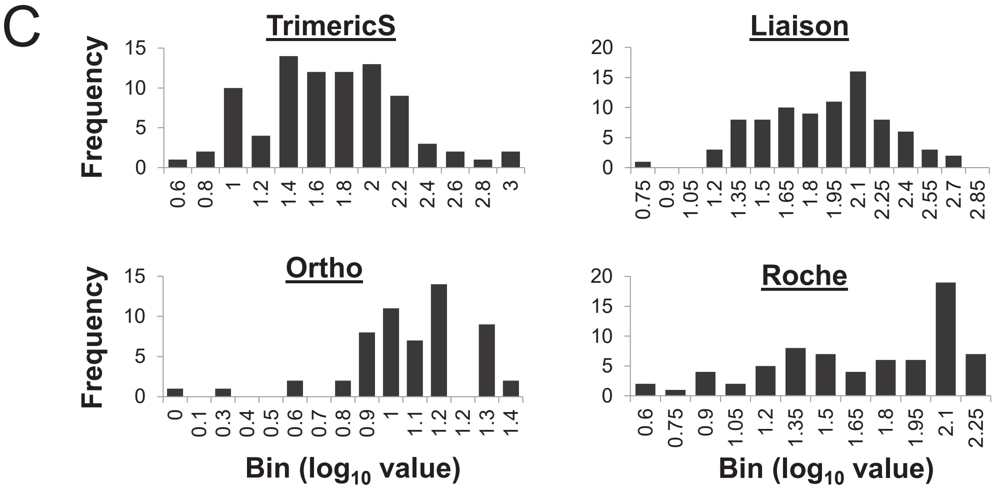

Supplemental Figure S1. Measurements of spike-targeting antibodies in 85 convalescent samples using immunoassays that apply different components of SARS-CoV-2 as capture antigens. **(A)** Distribution of the time interval between the PCR test indicating infection by SARS-CoV-2 and collection time of the samples used for this study. **(B)** Normality tests of immunoassay values. The Shapiro-Wilk test was performed for the immunoassay values and for the log_{10}-transformed immunoassay values. The null hypothesis for this test is that the data are normally distributed. *P* values lower than 0.05 indicate that the null hypothesis is rejected. **(C)** Distribution of immunoassay values measured for 85 convalescent using commercial tests.

Supplemental Figure S2

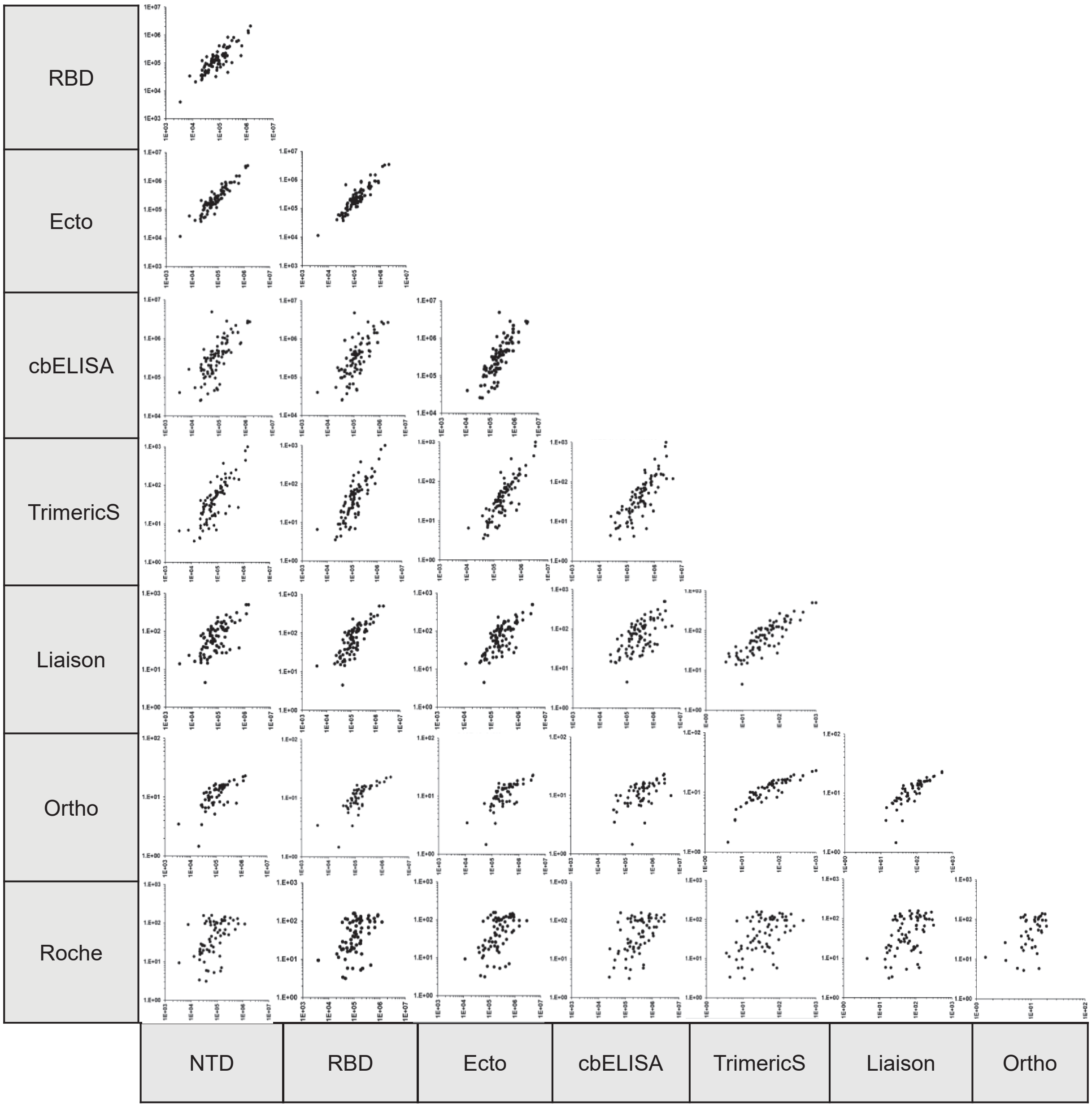

Supplemental Figure S2. Comparison of the log_{10}-transformed values measured in eight immunoassays for 85 convalescent serum and plasma samples. Values in the commercial assays are expressed in arbitrary units (AUs) whereas values in ELISAs are expressed in relative light units (RLUs). Values in the x and y axes are shown in log_{10} scale. Correlation coefficients are shown in **Fig. 1C**.

Supplemental Figure S3

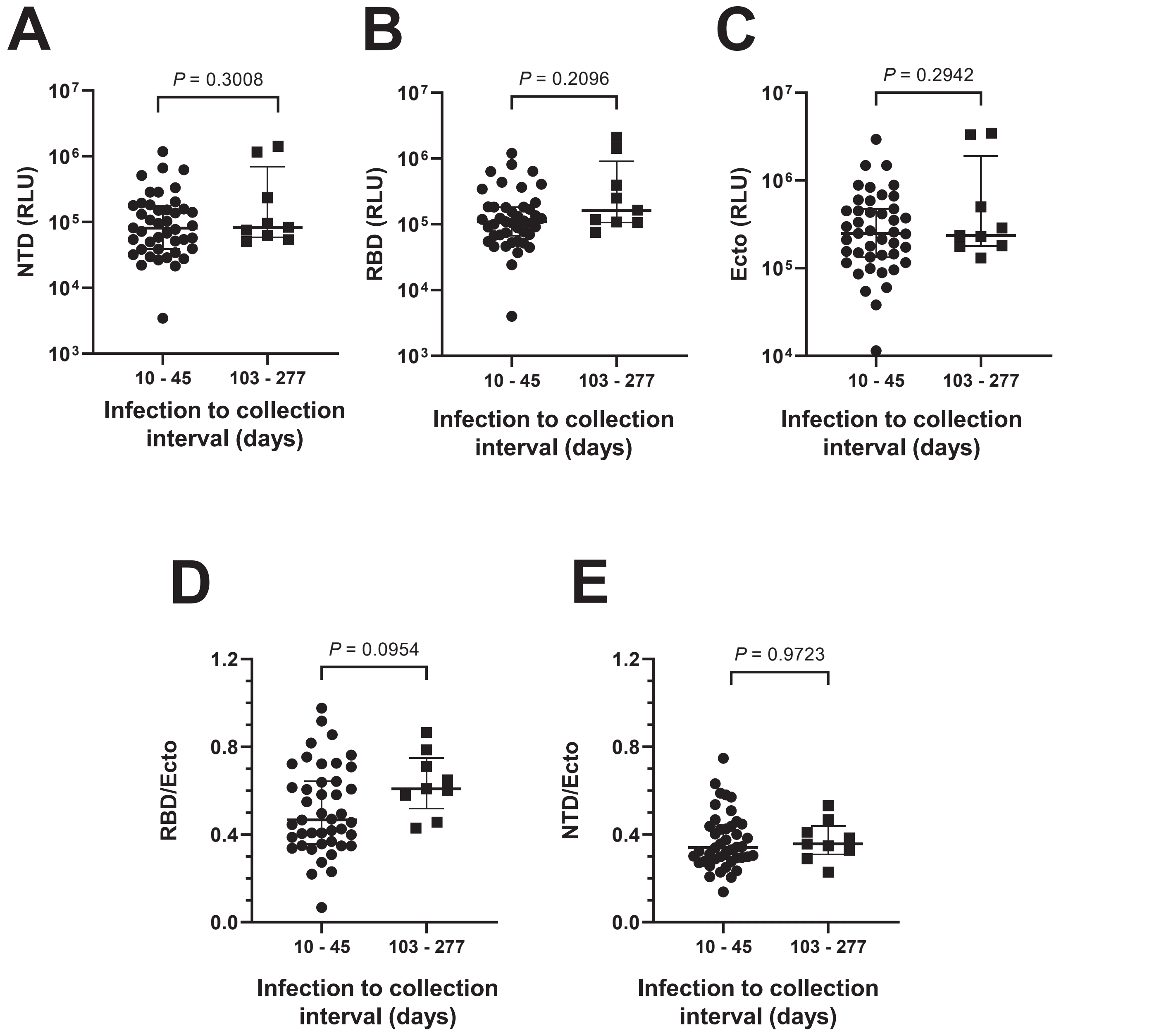

Supplemental Figure S3. Relationship between timing of sample collection and target specificity of the antibody response. The interval (in days) between the PCR-positive test indicating SARS-CoV-2 infection and the time of plasma or serum collection for these studies was determined. **(A-C)** Immunoassay values are compared between samples with an interval of 10-45 days (n=43), and samples with an interval of 103-277 days (n=9). **(D,E)** Comparison of the RBD/Ecto or NTD/Ecto ratios for samples collected after the indicated intervals from detection of infection by PCR. *P* Value, two-tailed test.

Supplemental Figure S4

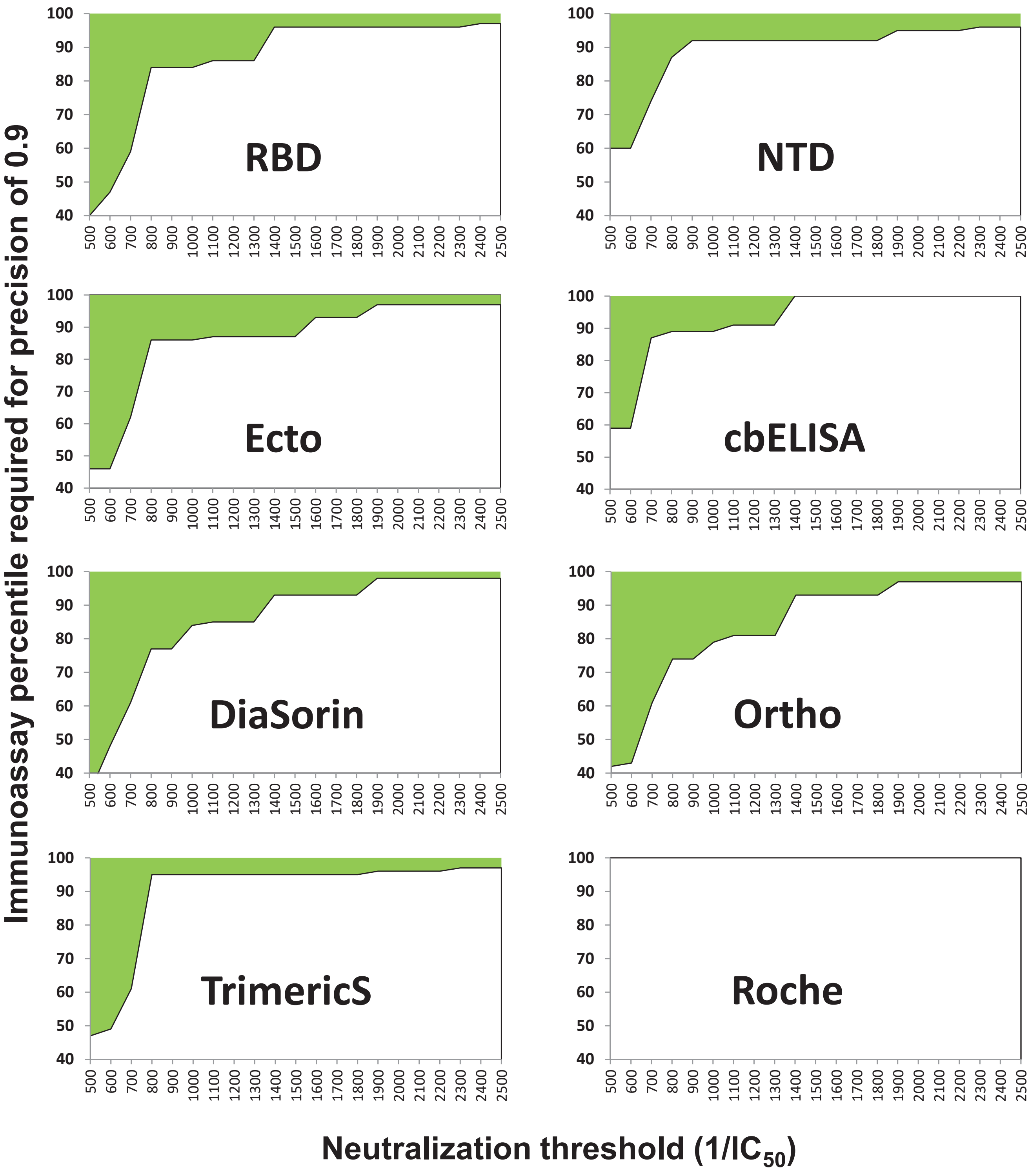

Supplemental Figure S4. Immunoassay percentiles required to predict neutralization at the indicated thresholds with a precision of 0.9. The shaded area describes the combination between neutralization thresholds and sample immunoassay percentiles that allow prediction with a precision of 0.9 or higher. The Roche test did not achieve a precision of 0.9, and thus a value could not be computed for this assay.

Supplemental Figure S5

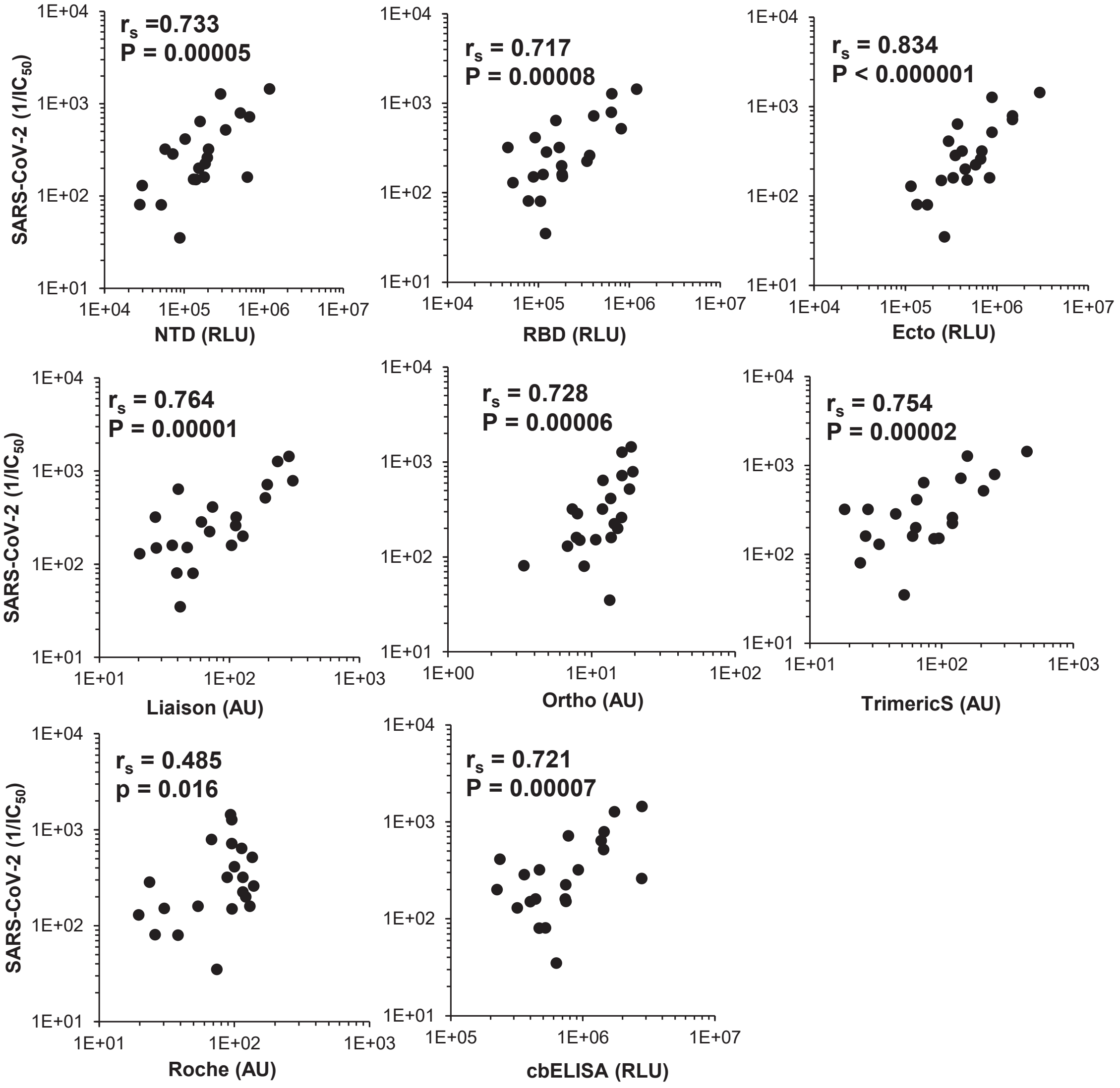

Supplemental Figure S5. Relationships between immunoassay values of COVID-19 convalescent samples and their neutralization of replicative SARS-CoV-2. r_S, Spearman correlation coefficient. *P* value, two-tailed test.

Supplemental Figure S6

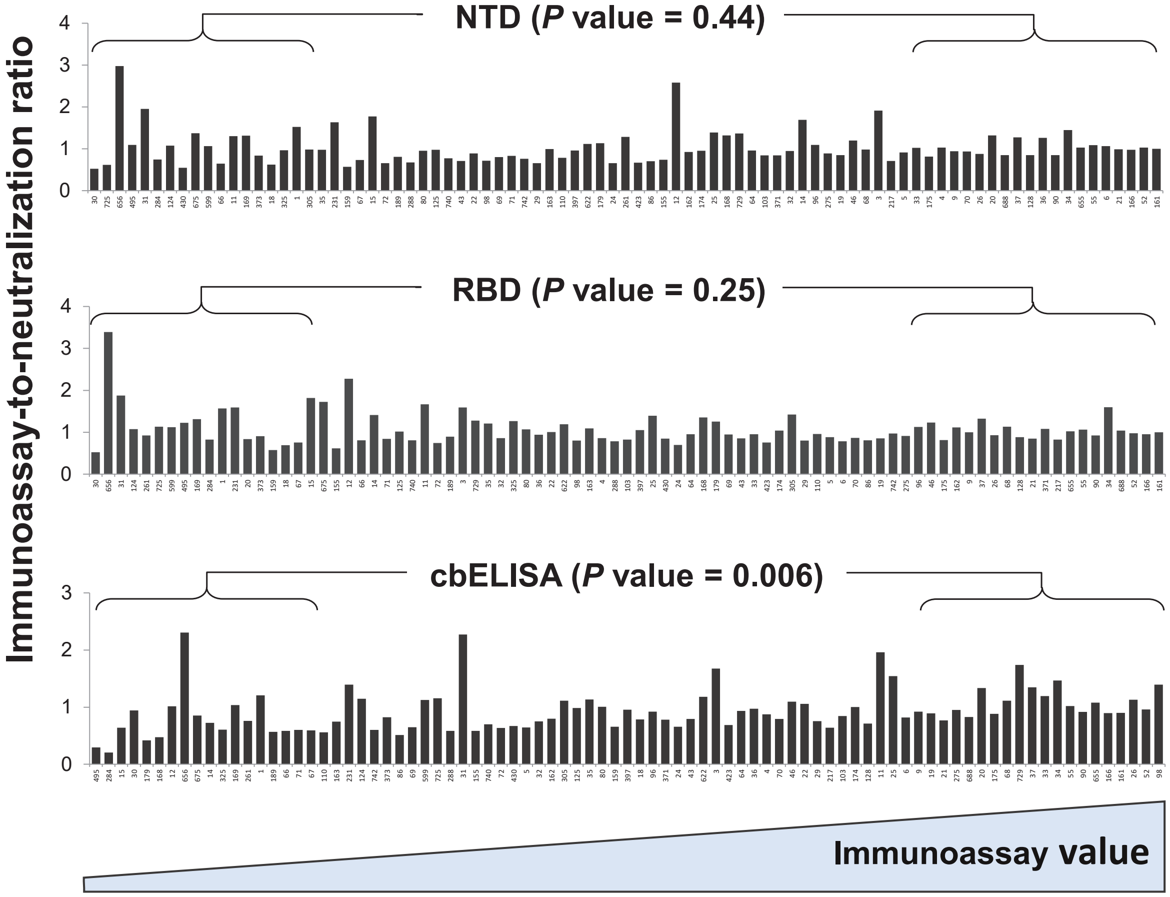

Supplemental Figure S6. Relationship between the level of spike-specific antibodies in convalescent samples and their relative neutralization potency. IC_{50} values of convalescent samples and their immunoassay values were log-transformed and adjusted to a scale of 0.1 to 1. For each sample, the ratio between the immunoassay value and the IC_{50} value was calculated and shown. Samples are ordered by increasing immunoassay values from left to right. The ratios calculated for the 20 samples with the lowest immunoassay values and the 20 samples with the highest immunoassay values were compared using an unpaired T test; the *P* values for a two-tailed test are indicated.
